# Observation of super-Alfvénic slippage of reconnecting magnetic field lines on the Sun

**DOI:** 10.1038/s41550-024-02396-4

**Published:** 2024-10-18

**Authors:** Juraj Lörinčík, Jaroslav Dudík, Alberto Sainz Dalda, Guillaume Aulanier, Vanessa Polito, Bart De Pontieu

**Affiliations:** 1 Bay Area Environmental Research Institute, NASA Research Park, Moffett Field, CA USA; 2 Lockheed Martin Solar and Astrophysics Laboratory, Palo Alto, CA USA; 3 Astronomical Institute of the Czech Academy of Sciences, Ondřejov, Czech Republic; 4 Sorbonne Université, Observatoire de Paris – PSL, École Polytechnique, Institut Polytechnique de Paris, CNRS, Laboratoire de physique des plasmas (LPP), Paris, France; 5 Rosseland Center for Solar Physics, University of Oslo, Blindern, Norway; 6 Institute of Theoretical Astrophysics, University of Oslo, Blindern, Norway; 7 Department of Physics, Oregon State University, Corvallis, OR USA

**Keywords:** Solar physics, Astrophysical magnetic fields, Astrophysical plasmas

## Abstract

Slipping motions of magnetic field lines are a distinct signature of three-dimensional magnetic reconnection, a fundamental process driving solar and stellar flares. While being a key prediction of numerical experiments, the rapid super-Alfvénic field line slippage driven by the ‘slip-running’ reconnection has remained elusive in previous observations. New frontiers into exploring transient flare phenomena were introduced by recently designed high cadence observing programs of the Interface Region Imaging Spectrograph (IRIS). By exploiting high temporal resolution imagery (~2 s) of IRIS, here we reveal slipping motions of flare kernels at speeds reaching thousands of kilometres per second. The fast kernel motions are direct evidence of slip-running reconnection in quasi-separatrix layers, regions where magnetic field strongly changes its connectivity. Our results provide observational proof of theoretical predictions unaddressed for nearly two decades and extend the range of magnetic field configurations where reconnection-related phenomena can occur.

## Abstract (web summary)

High-resolution solar flare observations captured rapid brightening in the Sun’s lower atmosphere. Their speeds of thousands of kilometres per second provide a missing piece of evidence for magnetic reconnection in three dimensions.

## Main

Magnetic reconnection, the change of connectivity of individual magnetic field lines to a lower energy state, is a fundamental physical process present throughout astrophysical and laboratory plasmas^1^. Magnetic reconnection is widely believed to drive solar and stellar flares and eruptions, violent releases of magnetic energy from the atmosphere of the Sun and major constituents of space weather. Observations of these phenomena are, however, often limited by instrumental time resolution, challenging the validation of current models of reconnection in the complex three-dimensional (3D) reality.

Magnetic energy released during reconnection is converted into acceleration of charged particles and plasma heating. The released energy is propagated to the dense lower atmosphere of the Sun, where it is dissipated^2^. There, bright flare ribbons are formed at footpoints of reconnecting magnetic field lines. The ribbons are usually elongated structures, routinely observed in visible, ultraviolet (UV), and extreme ultraviolet (EUV) lines emitted by the solar chromosphere and the transition region^3^. They correspond to footpoints of reconnecting field lines forming arcades of hot (*T* ≈ 10 MK) flare loops filled with plasma evaporated from the chromosphere^4^. Ribbons are dynamical structures, typically exhibiting ‘separation’ motions^5,6^, or spreading away from the polarity inversion line (PIL). Rather than being uniform structures, ribbons are composed of individual flare kernels^7^, minor brightenings often observed to exhibit motions along the ribbons. Combined observational and modelling efforts^8–10^ showed that kernel motions are signatures of magnetic slipping reconnection^11,12^. In addition to kernel motions, slipping reconnection has been identified as the mechanism leading to slipping motions (‘slippage’) of active region loops^13^, flare loops emanating from kernels^8,14,15^, as well other flare-related phenomena^16,17^. Flare loops were found to typically exhibit slipping speeds *v*_slip_ < 10^2^ km s^−1^ (refs. ^8,15,18^), while kernels can be faster. The highest *v*_slip_ reported so far was up to ~450 km s^−1^ (ref. ^10^).

Unlike true plasma motions, slipping motions are only apparent motions, arising as a consequence of the energy deposition and thermal response of plasma to the sequential change of connectivity in the quasi-separatrix layers (QSLs^19–21^). QSLs are central features of 3D magnetohydrodynamic (MHD) simulations of flares and eruptions. They generalize concepts of magnetic reconnection in solar^22–24^ and laboratory^25^ plasmas, as well as in plasmas elsewhere in the Universe. During reconnection, magnetic field lines within QSLs change their connectivity sequentially from one to another, characterized by their apparent slippage along footprints of QSLs, which in observations correspond to flare ribbons^26,27^.

MHD modeling of solar flares and eruptions^12,28–30^ has shown that the slipping motions can be either sub- or super-Alfvénic, depending on whether *v*_slip_ surpasses the coronal Alfvén speed *c*_A_. Considering typical *c*_A_ vaules in the solar corona^31^, the slip-running reconnection should be manifested in rapid motions at *v*_slip_ of several 10^2^ and 10^3^ km s^−1^. However, the slipping speeds observed so far systematically fall into the sub-Alfvénic regime.

The lack of evidence for slip-running reconnection challenges fundamental concepts of 3D reconnection in QSLs. QSLs are generally defined as regions with high gradients of magnetic connectivity, which however remains continuous, unlike in the presence of null points known from the two-dimensional standard flare model^32–35^. The distortion of connectivity can be described in terms of the norm *N*, a dimensionless quantity, as regions where *N* ≫ 1 (ref. ^36^). It has been shown through 3D MHD extensions to the standard flare model^30^ that *N* can be estimated using the ratio of *v*_slip_ and the velocity of QSL footprints (*v*_QSL_ or *v*_⊥_) away from the PIL. This motion in observations corresponds to the ribbon separation, and the relation *N* = *v*_slip_/*v*_⊥_ relates *v*_slip_ and *v*_⊥_ measured in conjugate ribbons^18^. Previous attempts to estimate this quantity led to *N* ⪅ 7 (ref. ^18^), too low for the condition (*N* ≫ 1) for the QSL reconnection to be met.

The time resolution (cadence) of data is of paramount importance for resolving the slip-running reconnection in QSLs. It has been suggested that detection of fast slipping motions driven by slip-running reconnection might not be possible in observations acquired at cadences on the order of 10 s, typically employed in the past^10,18^. Here, we overcome this issue by exploiting unique capabilities of the Interface Region and Imaging Spectrograph (IRIS^37^) to probe the dynamics of slipping kernels in observations of a confined flare acquired at a ~2 s cadence. We demonstrate that kernel slippage does occur at speeds on the same order of magnitude as the coronal Alvén speeds, thus providing a key critical observation hitherto missing. Our observations are consistent with field line reconnection in QSLs, extending the range of magnetic configurations where magnetic field lines reconnect beyond null points. These results link predictions of MHD models of magnetic reconnection in 3D and modern-era observations of solar flares and provide insight into how the magnetic energy is released during solar flares.

## Results

### Overview of the event

**Fig. 1:**
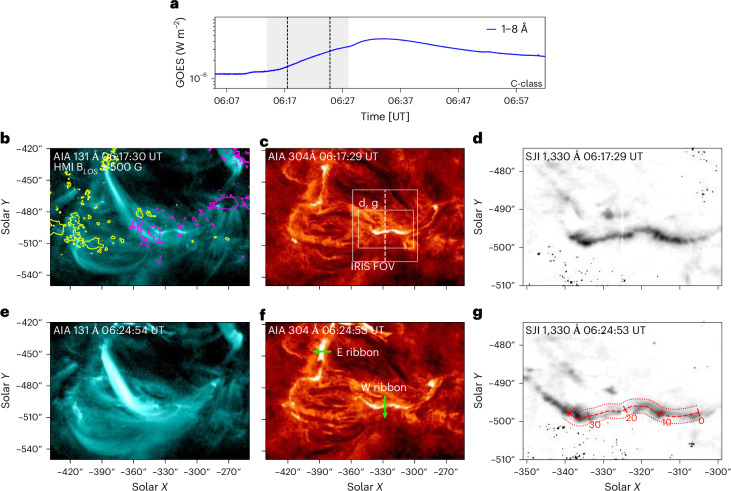
Context observations of the 2022 September 25 flare. **a**, The soft X-ray flux in the 1–8 Å channel of the GOES satellite. The grey shaded area indicates the time period under study. **b**–**g**, Depictions of the flare at the first (**b**−**d**) and second (**e**−**g**) instants marked by dashed lines in **a**, showing flare loops observed in AIA 131 Å (**b** and **e**), the ribbons in AIA 304 Å (**c** and **f**) and the western ribbon observed in IRIS SJI 1,330 Å (in reversed intensity) (**d** and **g**). **c**, Rectangles indicate the field of view in panels **d** and **g**. The contours in **b** indicate the photospheric magnetic field *B*_LOS_ saturated to ±500 G, while yellow and magenta indicate positive and negative magnetic flux, respectively. The arrows in **f** and **g** delineate the artificial cuts across and along the ribbons, respectively, used to construct time–distance diagrams. The brightest emission is plotted in white color in **b**, **c**, **e** and **f**. Black color represents the highest intensities in **d** and **g**. SJI observations in **d** and **g** are also presented in Extended Data Fig. 5 and their animated version in Supplementary Video 3. FOV, field of view.

**Extended Data Fig. 1:**
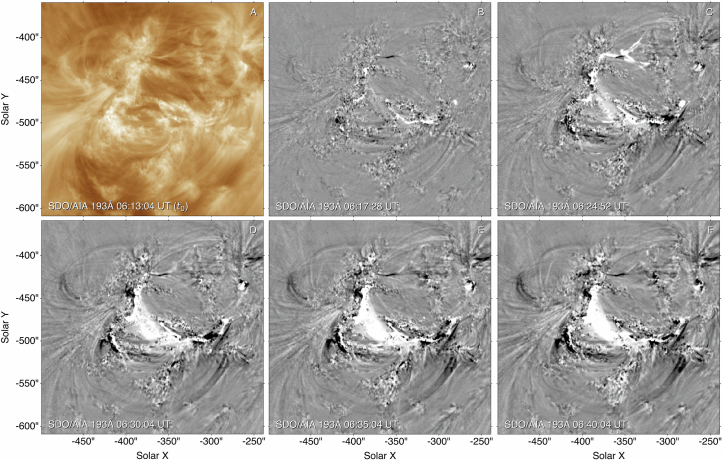
Evolution of coronal structures during the flare. Panel **a** shows AIA 193 Å observations of the NOAA 13107 active region during the onset of the flare (*t*_0_). Panels (**b** – **f**) contain base difference images produced by subtracting data observed at times showed therein from the initial snapshot plotted in panel **a**. Animated version of the AIA 193 Å base difference observations is presented in Supplementary Video 1.

**Extended Data Fig. 2:**
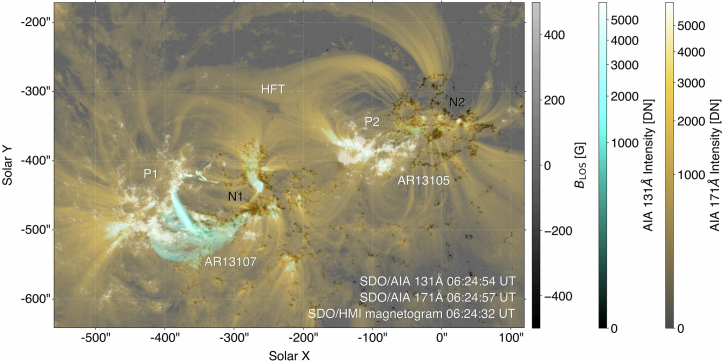
Observations of active regions associated to the flare. The background image presents the distribution of the line-of-sight magnetic field (*B*_LOS_) observed by HMI. The active region NOAA 13107 is located in the south-east. Hot flare loops observed in AIA 131 Å (cyan), are visible between the two major concentrations of positive (P1, white) and negative (N1, black) polarities of this active region. The NOAA 13105 active region is present in the north-west. The polarities comprising the two active regions, as well as the two regions 13105 and 13107, are connected by coronal loops observed in AIA 171 Å (yellow). Supplementary Video 2 presents an animated version of these observations.

The flare under study originated in the National Oceanic and Atmospheric Administration (NOAA) 13107 active region on 2022 September 25. It was observed by multiple space-borne instruments, with key observations made by the IRIS satellite in one of its newly designed high-cadence observation programs (Methods). Context observations of this event from the Geostationary Operational Environmental Satellite (GOES) and the Solar Dynamics Observatory (SDO^38^) are presented in Fig. 1a–g. The time evolution of the soft X-ray flux in the 1–8 Å channel of GOES is shown in Fig. 1a. The flare was of class C4.2 and started after 06:10 UT and peaked between 06:32 and 06:35 UT, with gradual phase lasting until ~07:50 UT. The flare was of a confined nature. EUV observations in the 193 Å passband of SDO/Atmospheric Imaging Assembly (AIA)^39^ (Extended Data Fig. 1b, Supplementary Video 1) did not reveal any associated erupting material. The magnetic structure of AR 13107 is indicated in Fig. 1b using contours corresponding to the line of sight (LOS) component of the magnetic field strength *B*_LOS_ ± 500 G as observed by the SDO/Helioseismic and Magnetic Imager (HMI)^40^ instrument. The magnetic environment of this active region is also presented in Extended Data Fig. 2 and Supplementary Video 2 and further discussed in Supplementary Section 1.1.

**Extended Data Fig. 3:**
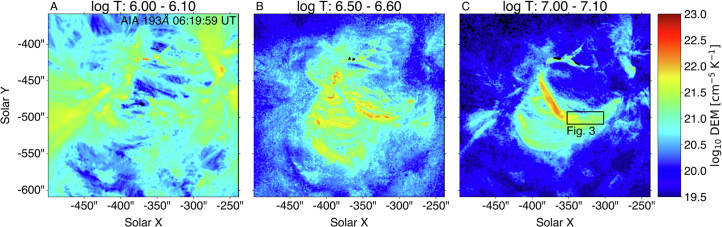
Temperature structure of the flare. Map of DEM recovered using snapshots in the six filter passbands of AIA (**Temperature diagnostics**) at ≈ 06:20 UT at log *T* [K] = 6.0 − 6.1 (panel **a**), log *T* [K] = 6.5 − 6.6 (panel **b**), and log *T* [K] = 7.0 − 7.1 (panel **c**).

**Extended Data Fig. 4:**
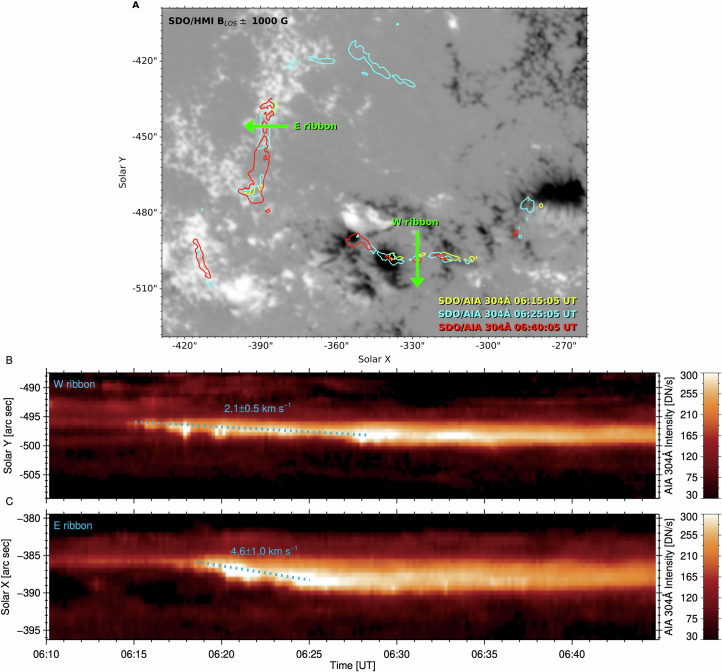
Ribbon separation during the flare. Panel **a** shows the distribution of *B*_LOS_ saturated to ± 1000 G as observed by the SDO/HMI. The colored contours correspond to AIA 304 Å passband intensity of 500 DN and capture the evolving ribbons in three different instants. Panels **b** and c detail time-distance diagrams capturing the separation motion of the western and eastern ribbon, respectively. The diagrams were constructed using the artificial cuts (indited as green arrows) in panel **a** along the direction of the ribbon motion.

The second and third rows of Fig. 1 detail the flare at 06:17:30 UT and 06:24:55 UT during its impulsive phase (Fig. 1a, black vertical lines). AIA 131 Å observations (Fig. 1b,e) show the presence of hot flare loops with characteristic temperatures of about log(*T* [K]) ≈ 7, determined via differential emission measure (DEM) analysis (Methods and Extended Data Fig. 3). Footpoints of these hot flare loops correspond to a pair of flare ribbons, one of which was positioned to the east (‘E ribbon’) and the other positioned to the west (‘W ribbon’). The ribbons, as observed in AIA 304 Å, are shown in Fig. 1c,f. The ribbons exhibited slow separation, that is, motion in the direction perpendicular to the ribbons, indicated using the green arrows in Fig. 1f and Extended Data Fig. 4. The separation speed of the W ribbon was found to be *v*_⊥_ = 2.1 km s^−1^. The conjugate E ribbon exhibited slightly faster movement at *v*_⊥_ = 4.6 km s^−1^. Full analysis of ribbon separation dynamics is presented in Supplementary Section 1.3.

### Slit-jaw imager observations of fast kernel slippage

**Fig. 2:**
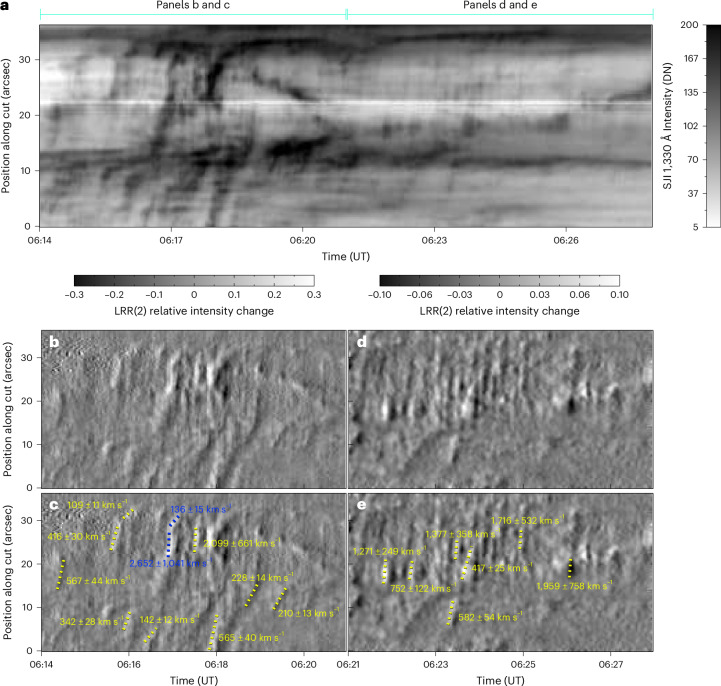
SJI 1,330 Å time–distance diagrams depicting the motions of the apparently slipping kernels along the ribbon. **a**, The motion of kernels during the entire time period between 06:14 UT and 06:28 UT analysed in this work. DN, data number. **b**–**e**, Time–distance diagrams constructed using LRR(2) data (Methods) for 06:14–06:21 UT (**b**) and 06:21–06:28 UT (**d**), and with dotted coloured lines (and captions) showing linear fits to traces (and respective velocities) imprinted by apparently slipping flare kernels (**c** and **e**). LRR(2), LRR method applied to two images in a sequence.

**Extended Data Fig. 5:**
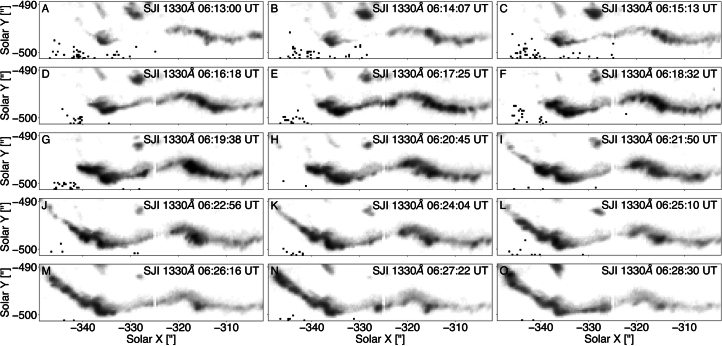
SJI 1330 Å observations of the western ribbon during the flare. The observations cover the period between 06:13 UT (panel a) and 06:28:30 UT (panel o). Supplementary Video 3 presents animated version of these observations.

The IRIS slit-jaw imager (SJI) captured the western ribbon located in the concentrations of the negative polarity flux. Figure 1d,g shows a detailed view of this ribbon observed in the SJI 1,330 Å passband. This ribbon was composed of bright kernels, visible as patches of enhanced emission, many of which exhibit apparent motions along the ribbon (Extended Data Fig. 5 and Supplementary Video 3). The kernel motions imprint elongated traces visible throughout the time–distance diagram (Fig. 2a) constructed along the artificial cut through the SJI 1,330 Å data denoted by the red arrow in Fig. 1g. Many of the kernel traces are faint. To enhance the intensity changes associated with these kernels, the IRIS/SJI data were processed using the log-running-ratio (LRR) method with a difference of two frames (Methods). The time–distance diagrams produced using the processed data (Fig. 2b–e) were then used to measure the apparent slipping velocities of kernels. Linear fits to traces of slipping kernels (Methods) are indicated using colored dotted lines in Fig. 2c,e. The fits indicate that the kernels were slipping at a wide range of velocities, between *v*_slip_ = 109 ± 11 km s^−1^ and 2,652 ± 1,041 km s^−1^. The vast majority of kernel motions were directed toward the eastern ribbon, though several instances of oppositely oriented slippage were also observed. Multiple kernels exhibit *v*_slip_ on the order of 10^3^ km s^−1^, corresponding to the typical Alfvén speeds in the solar corona (Supplementary Section 1.2) consistent with the slip-running reconnection. The norm *N* estimated using the observed *v*_slip_ (109–2,652 km s^−1^) combined with the *v*_⊥_ = 4.6 km s^−1^ of the E ribbon (Supplementary Section 1.3) ranges between 24 ± 6 and 577 ± 259. The high (*N* ≫ 1) values are representative of high gradients of magnetic connectivity within QSLs, as described above.

**Fig. 3:**
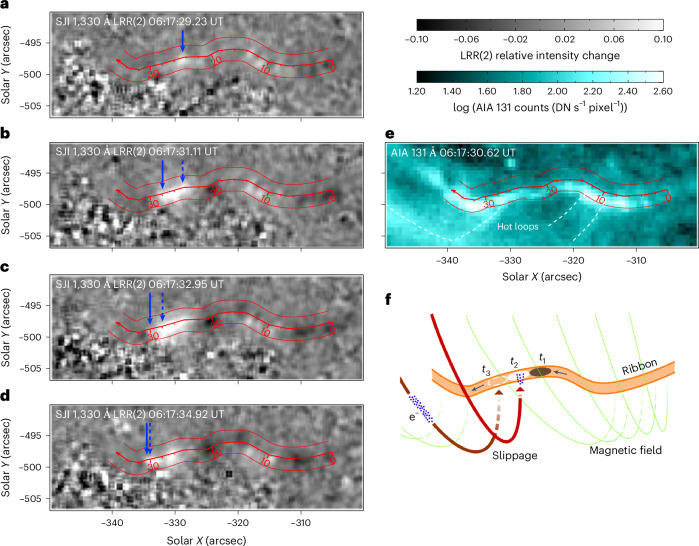
The apparent slipping motion of a selected kernel. **a**–**d**, The kernel motion along the ribbon observed in SJI 1,330 Å LRR(2) images as time increases from 06:17:29.23 (**a**) to 06:17:31.11 (**b**) to 06:17:32.95 (**c**) to 06:17:34.92 (**d**). Solid blue arrows denote the location of the kernel front along the direction of motion, while dashed arrows show its location in the last SJI image. The cut used to study the kernel motions along the ribbon (Results) is shown as a red curve (with distance indicated), and the cut width is denoted by thin red lines. **e**, The same region observed by AIA 131 Å, detailing hot flare loops emanating from kernels. **f**, A cartoon providing a simplified description of the kernel motion and emission as a consequence of the slipping reconnection. LRR(2), LRR method applied to two images in a sequence.

The motion of the fastest kernel, highlighted using the blue fits in Fig. 2c, is further detailed in Fig. 3a–d. There, time increases from top (Fig. 3a) to bottom (Fig. 3d). The solid blue arrows mark the approximate location of the front side of the kernel along the direction of its motion, while the dashed ones indicate its approximate location in the previous SJI snapshot. The motion of the analysed kernel consisted of two episodes. The first, fast episode of the kernel’s motion with *v*_slip_ = 2,652 ± 1,041 km s^−1^ was visible in three snapshots and is detailed in Fig. 3a–c. The second episode consisted of kernel deceleration with terminal *v*_slip_ = 136 ± 15 km s^−1^, captured in Fig. 3c,d. This deceleration occurred as the kernel entered a bright part of the ribbon roughly at the 30″ coordinate of the cut (Fig. 1g), coincident with a strong concentration of photospheric magnetic flux. Physical factors determining the magnitude and variability of *v*_slip_ are discussed in Supplementary Section 1.5.

AIA 131 Å observation of the same region, plotted in Fig. 3e, reveals several faint hot flare loops (white dashed lines) anchored in the ribbon where the slip-running kernel motions occurred. Although the cadence of AIA is not sufficiently high to capture these fast motions, its observations indicate that signatures of the slipping reconnection were still associated with hot flare emission, in agreement with previous studies^8,10,18^.

A simplified schematic picture of the apparent kernel motion is provided in Fig. 3f. This cartoon depicts the flare ribbon (orange) and a kernel slipping in the direction indicated by the black arrows. The kernel observed in the present instant *t*_2_ (white ellipse) is visible as a result of energy deposition, presumably caused by the accelerated electrons (blue dots) that propagate along the slipping magnetic field lines (example shown in red). The black ellipse represents the former kernel location during the instant *t*_1_ where the energy deposition no longer continues. In the next snapshot (*t*_3_), the kernel becomes visible further along the ribbon (white dashed ellipse) as the electrons accelerated by continuing reconnection precipitate along the slipping field lines further along the ribbon (example shown in brown). The entire time sequence *t*_1_–*t*_3_ represents the apparent slipping motion of the kernel.

**Extended Data Fig. 6:**
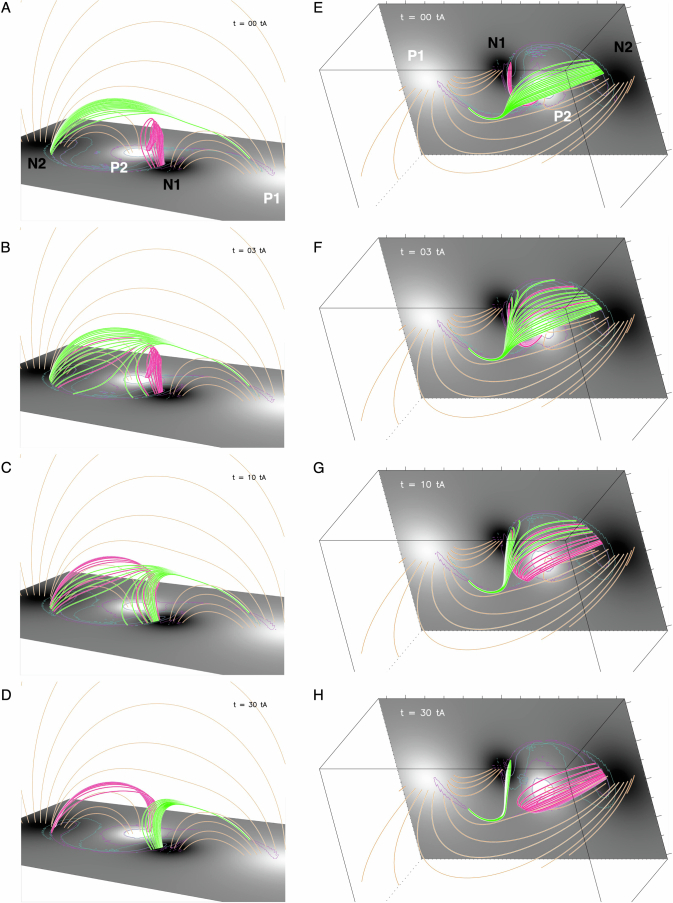
Slip-running reconnection in a 3D MHD model of a confined flare. Sequential change of connectivity between green and magenta field lines captured during four instants (*t* = 0, 3, 10, 30 *t*_A_) in the simulation described in 3D MHD model of field line slippage. Black and white patches at the base of the domain represent negative (N1, N2) and positive (P1, P2) polarity flux concentrations saturated to *B*_z_ = 0 = ± 25 (in code units). Pink and cyan contours encompass vertical electric current concentrations with *J*_z=0_ = ± 0.3 (in code units) along QSL footprints. Panels a - d and e - h present the simulation output in the four instants from two different vantage points.

Coincidentally, the flare analysed here occurred in a magnetic environment (Supplementary Section 1.1) reminiscent of that used in the original 3D MHD numerical experiment^12^ that predicted the super-Alfvénic field line slippage. This model is illustrated in Extended Data Fig. 6, where the initially long field lines (plotted in green) reconnect with conjugate (magenta) field lines, eventually turning to field lines similar to flare loops visible, for example, in AIA 131 Å (for example, Fig. 1e). This evolution occurs through slip-running reconnection, with the footpoints of the green lines slipping along the negative-polarity N1 in the middle of the overall quadrupolar configuration, as observed. A comparison of this vintage simulation with the modern observations, detailed in Supplementary Section 1.4, allowed us to put the fast kernel slippage observed by IRIS in a broader context of the full quadrupolar environment of this confined flare.

### Importance of cadence for resolving fast kernels

The observed kernel velocities notably exceed the sub-Alvénic slippage reported in the past, often relying on AIA observations (as described above) acquired at a cadence of 12 s (304 Å passband) or 24 s (1,600 Å passband). Considering that the cadence of the high-cadence IRIS/SJI observations substantially surpasses that of AIA 304 Å and 1,600 Å, by factors of 6 and 12, respectively, it is worth investigating how the instrument cadence as well as spatial resolution impact the detection of high-velocity kernels.

**Fig. 4:**
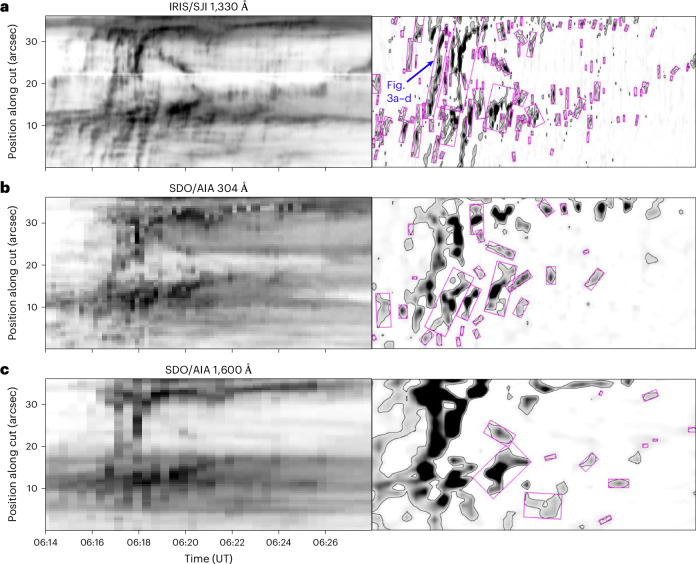
Time–distance diagrams detailing the slipping motion of kernels as observed by different instruments and filter passbands. Time–distance diagrams obtained using data from SJI 1,330 Å (**a**, same as Fig. 2a), AIA 304 Å (**b**) and AIA 1,600 Å (**c**). Left: time–distance diagrams. Right: after processing using the Sobel operator for the detection of edges along the vertical direction. Magenta boxes mark stripes indicating moving features detected using the computer vision algorithm described in Methods. The black color in the images corresponds to the brightest emission.

The left column of Fig. 4 shows time–distance diagrams detailing kernel motions as observed by instruments (filter passbands) with different temporal and spatial resolutions. In addition to the original SJI 1,330 Å time–distance diagram (Fig. 4a), this figure contains time–distance diagrams produced using the AIA 304 Å (Fig. 4b) and 1,600 Å data (Fig. 4c) along the same cut (Fig. 1g, red arrow). The effects of the instrument cadence and resolution have been quantified via a computer vision technique (Methods). To increase the efficiency of the algorithm for the detection of features corresponding to kernel traces, the time–distance diagrams were processed using the Sobel operator for the detection of edges (Fig. 4, right column). Traces of slipping kernels detected by this method are highlighted by the magenta frames overplotted in the right column of Fig. 4.

The number of computer-detected features *n*_f_ along with the mean absolute slipping velocities $\left\langle | {v}_{{\rm{slip}}}| \right\rangle$ and standard deviations ${\sigma }_{| {v}_{{\rm{slip}}}| }$ derived from the feature orientation are summarized in Table 1. This comparative analysis is indicative of a notable and progressive decrease of *n*_f_ between the time–distance diagrams produced using IRIS (Fig. 4a) and AIA data (Fig. 4b,c) with lower temporal and spatial resolution. The *n*_f_ in the SJI 1,330 Å time–distance diagram exceeds that detected in the AIA 304 and 1,600 Å images by an order of magnitude, except for cases where the detection threshold is high (Methods).

**Table 1:** Number of features *n*_f_ detected in the time–distance diagrams by the computer vision technique

Intensity threshold (% of ${I}_{\max }$)	10			30			50			90		
	*n*_f_	$\left\langle | {v}_{{\rm{slip}}}| \right\rangle$	${\sigma }_{| {v}_{{\rm{slip}}}| }$	*n*_f_	$\left\langle | {v}_{{\rm{slip}}}| \right\rangle$	${\sigma }_{| {v}_{{\rm{slip}}}| }$	*n*_f_	$\left\langle | {v}_{{\rm{slip}}}| \right\rangle$	${\sigma }_{| {v}_{{\rm{slip}}}| }$	*n*_f_	$\left\langle | {v}_{{\rm{slip}}}| \right\rangle$	${\sigma }_{| {v}_{{\rm{slip}}}| }$
SJI 1,330	112	1,198	1,045	67	1,058	835	53	1,096	1,025	29	1,122	1,401
AIA 304	25	358	690	21	94	60	16	89	63	11	96	48
AIA 1,600	12	9	11	8	16	15	9	9	7	1	4	-
SJI d. TRACE (2 s)	95	2,291	1,298	60	1,943	1,209	43	1,969	1,304	29	2,295	1,780
SJI d. HI1	92	472	494	65	272	218	40	360	333	24	315	376
SJI d. HI2	34	96	85	38	158	378	29	89	83	20	92	75

Average kernel velocities $\left\langle | {v}_{{\rm{slip}}}| \right\rangle$ and the standard deviations ${\sigma }_{| {v}_{{\rm{slip}}}| }$ (in kilometres per second), calculated using the average orientation of the detected features, are indicated. Different columns of the table designate the three quantities determined using time–distance diagrams saturated to selected intensity thresholds (10–90% of the maximal intensity ${I}_{\max }$).

The range of ∣*v*_slip_∣ calculated using the orientations of features detected in the SJI 1,330 Å diagram is consistent with that resulting from the manual fitting (Supplementary Section 1.6.1). The mean kernel velocities $\left\langle | {v}_{{\rm{slip}}}| \right\rangle$ determined via the computer vision method consistently surpass 10^3^ km s^−1^ (Table 1) and thus confirm our discovery of super-Alfvénic slipping motions. In addition, the $\left\langle | {v}_{{\rm{slip}}}| \right\rangle$ corresponding to the features detected in the SJI time–distance diagram are 1–2 orders of magnitude higher than those obtained using the AIA 304 Å and 1,600 Å data, respectively. By implementing various degradations of the SJI 1,330 Å observations (Supplementary Section 1.6.2), we found that a time resolution of about 4 s is essential for detection of the fastest (∣*v*_slip_∣ > 10^3^ km s^−1^) kernels. While the role of cadence is critical, the detection of super-Alfvénic kernel motions is also influenced by additional factors including spatial resolution as well as the spectral content of the observations. Signatures of kernel motions detected in narrow-band observations with contributions from the transition region, such as those analysed here, may differ from those visible in the chromosphere or the photospheric continuum formed below the transition region.

## Discussion

This study presents an analysis of the dynamics of flare ribbon kernels exhibiting apparent slipping motions in IRIS observations of a confined C4-class flare from 2022 September 25. The exceptionally high spatial (0″.33) and temporal (1.9 s) resolutions of IRIS played an essential role in the discovery of fast kernel motions with speeds of up to *v*_slip_ ≈ 2,600 km s^−1^ as measured using two independent methods. Exceeding previous measurements by an order of magnitude, these kernel slipping speeds correspond to and exceed the typical Alfvén speeds in the solar corona, indicating that the observed kernel slippage is a signature of slip-running reconnection.

Our analysis provides a key observable validating theoretical predictions that had remained unverified for nearly two decades. Thus, it consolidates slipping reconnection as a general process of magnetic energy release in solar flares and eruptions. The evidence for slip-running reconnection presented here complements earlier analyses of field line slippage manifested across a variety of solar transient phenomena, such as motions of flare loops and kernels^8,9,15,18^, elongation of flare ribbons^10^, pre-flare activity^16^ and the formation of sigmoidal active regions^17^ as well as slipping motions of coronal loops^13,41^ in non-flaring active regions.

The super-Alfvénic slipping motions are consistent with high distortion of magnetic connectivity within the flaring region. The higher limit of the norm *N* of field line connectivity, *N* > 500, estimated via the measurements of kernel and ribbon dynamics, is in a good agreement with the predictions of the 3D extensions to the standard flare model^30^. Our results therefore provide a direct link between observations and models of solar flares and eruptions based on 3D reconnection in QSLs outside of magnetic null points. Despite the absence of null points, the super-Alfvénic change of connectivity during slip-running reconnection maintains MHD systems behaving as in true null-point topologies^12^, extending the range of magnetic field configurations where reconnection-related phenomena develop.

## Methods

### Observations and data processing

In this study, we primarily analysed data acquired by the IRIS satellite. IRIS produces spectral observations of the Sun in two far-ultraviolet bands (1,331.6–1,358.4 Å and 1,380.6–1,406.8 Å) and one near-ultraviolet band (2,782.6–2,833.9 Å). IRIS also provides imaging observations via its SJI. The dataset utilized in this study contains observations in the SJI 1,330 Å passband, which is dominated by emission from the C ii 1,335 Å and 1,336 Å lines formed in the upper chromosphere with contributions from the transition region. SJI 2,796 Å observations dominated by the chromospheric Mg ii K line (2796 Å) emission are also available, however we opted to analyse the 1,330 Å data as they provide higher contrast between the emission of ribbon and the surrounding plasma. The size of an unbinned SJI pixel is 0″.167, which is about 120 km at the surface of the Sun, and the spatial resolution of IRIS is roughly 0″.33. The point spread function (PSF) of IRIS is limited by the Nyquist criterion, based on planetary transit data^42^. IRIS observed the event in one of its new high-cadence observing programs at an SJI cadence of ~1.9 s with ~0.3 s exposure times (in SJI 1,330 Å) and spatial binning of 2 × 2. Because of the short exposures, only a few counts were detected from regions surrounding the observed flare ribbon. Despite the presence of noise, using manual inspection of small-scale structures, we verified that the spatial resolution corresponds to the Nyquist criterion of two (super-)pixels. The level 2 science-ready IRIS data were handled using routines available within the SolarSoft package in the Interactive Data Language (IDL) as well as the ‘iris_lmsalpy’ and ‘irispy_lmsal’^43^ libraries in Python. Techniques used for further post-processing of SJI observations are detailed below.

The flare was also observed at multiple wavelengths by the AIA telescope on board the SDO spacecraft. The AIA produces full-Sun images with a pixel size of 0″.6, spatial resolution of 1″.5 and cadence of 12 s or 24 s, depending on the filter channel. The AIA observes the Sun in seven EUV and three UV passbands sensitive to plasmas radiating across a wide range of characteristic temperatures, from log(*T* [K]) = 3.7 to 7.2 (ref. ^44^). The 94 Å (log(*T* [K]) = 6.8), 131 Å (log(*T* [K]) = 5.6 and 7.0), 171 Å (log(*T* [K]) = 5.8), 193 Å (log(*T* [K]) = 6.2 and 7.3), 211 Å (log(*T* [K]) = 6.3) and 335 Å (log(*T* [K]) = 6.4) EUV passbands were used to investigate the temperature structure of the flare. In addition, we employed the AIA 131 Å data to study the emission of flare loops, AIA 304 Å (log(*T* [K]) = 4.7) and 1,600 Å (log(*T* [K]) = 5) passbands to analyse the emission of flare ribbons and finally the AIA 193 Å to analyse the evolution of plasma of the solar corona during the event. We also used measurements of the LOS component of the photospheric magnetic field (*B*_LOS_) from the HMI of the SDO mission.

The AIA observations were corrected for the differential rotation with the reference time set to 06:20 UT and deconvolved with the instrumental PSF. Data processing and visualization were carried out using the SunPy and SolarSoft libraries. To produce the composite image plotted in Extended Data Fig. 2, depicting data from the HMI, AIA 131 Å and 304 Å, we employed the screen blending operator provided in the ‘mplcairo’ back-end implementation for the ‘matplotlib’^45^ library in Python. To enhance the overall contrast of the image, we slightly shifted the conventional colour table used for plotting HMI data. For the same purpose, the AIA 131 Å and 171 Å snapshots were adjusted using gamma correction with *γ* = 0.3 and 0.4, respectively.

We found a minor misalignment between the SJI dataset and full-disk AIA observations. The shift between the SJI and AIA datasets was quantified by comparing locations of bright emission of the W ribbon as observed in the SJI 1,330 Å and AIA 304 Å as well as 1,600 Å snapshots taken at roughly 06:17 UT. A shift of *X* = − 2.2″, *Y* = −2.4″ (in helioprojective Cartesian system) was applied to the IRIS data to achieve coalignment between the two instruments. In addition, owing to a minor failure of the limb detection algorithm, the AIA 304 Å passband suffered from a pointing shift at roughly 06:00 UT of 2022 September 25 (AIA team, private communication). To coalign this passband with other AIA channels, we used a shift of *X* = −2″, *Y* = 2″ found upon comparison of AIA 304 Å and AIA 1,600 Å observations of ribbons.

### Time–distance diagrams

Speeds *v*_⊥_ of ribbon motion away from the PIL (separation) as well as the dynamics of slipping kernels observed along the W ribbon were studied using time–distance diagrams. The time–distance diagrams detailing the ribbon separation motion were produced along artificial cuts denoted using green arrows in Fig. 1f. The motion of kernels was studied via the artificial cut tracing the ribbon as observed in SJI 1,330 Å, indicated using the curved red arrow in Fig. 1g. Because of the slow, ~2 km s^−1^ separation motion of this ribbon (Results), we employed a half-width of the cut of 2″ (Fig. 1g, thin dotted lines). This width allowed us to use a single cut for the entire time period under study. The signal at a given position along the cut is a sum over the width of the cut. Since the ribbon is the only strongly emitting structure along the cut at all times, it always dominates.

Because many of the kernels are fast and their intensity is weak, we employed the LRR method to enhance faint moving structures in imaging observations^46^. The intensity of the *i*th image in a series is given by

1 $${\rm{LRR}}{(d)}_{i}=\log \left(\frac{{I}_{{\rm{SJI}},i}}{{I}_{{\rm{SJI}},i-d}}\right)\,,$$

where *d* is the difference of the time indices between the used frames. We found that *d* = 2 (that is, a difference of ~3.8 s) enhances the motion of kernels well while not leading to different kernel velocities (see below) when compared with *d* = 1. The disadvantage of the LRR method is that, in weak signal regions outside the ribbon, it produces strong spurious signal due to noise amplification. In practice, we find that, given the low exposure time of IRIS required for fast cadence, the LRR is useful only within the ribbon, as other areas are noise dominated (producing a checkered black and white pattern; Fig. 3a–d). For display purposes, this noise is suppressed in these images by 3 × 3 pixel boxcar smoothing. Note that the LRR saturation of ±0.1 and ±0.3 used in Fig. 3 corresponds to a change in *I*_SJI_ of about 25% and 100%, respectively.

In time–distance diagrams (Fig. 2), the apparently slipping flare kernels imprint elongated traces of enhanced intensities. The larger their inclination from the horizontal direction in the diagrams, the faster the kernel motion. Near-vertical traces are indicative of fast flare kernels, whereas horizontal ones are imprinted by slow or even stationary kernels. The velocities of the kernel’s apparent slipping motion *v*_slip_ are measured using spatial and temporal differences between the endpoints of linear fits to these traces, *v*_slip_ = (*s*_1_ − *s*_0_)/(*t*_1_ − *t*_0_). The maximal *v*_slip_ distinguishable in SJI 1,330 Å data, estimated by considering the longest (*s*_1_ − *s*_0_ ≈ 7″.5), near-vertical traces found in Fig. 2 was found to be roughly 6,000 km s^−1^. The uncertainty of the slipping velocity *σ*(*v*_slip_) was estimated using the equation^47^

2 $$\sigma ({v}_{{\rm{slip}}})=\sqrt{\frac{{\sigma }^{2}({s}_{0})+{\sigma }^{2}({s}_{1})}{{({t}_{1}-{t}_{0})}^{2}}+{v}_{{\rm{slip}}}^{2}\frac{{\sigma }^{2}({t}_{1})+{\sigma }^{2}({t}_{0})}{{({t}_{1}-{t}_{0})}^{2}}}\,,$$

where *σ*(*s*_0,1_) = 0″.167 and *σ*(*t*_0,1_) = 0.48 s are the uncertainties of the endpoints of the linear fits taken as a quarter of the spatial and temporal resolution^47^. These values provide an uncertainty in the resulting velocity that is in excellent agreement with the velocities obtained by repeated manual linear fitting of the kernel traces. In cases of fast kernel motions, the second fraction under the square root dominates the first one by several orders of magnitude, since it is proportional to ${({t}_{1}-{t}_{0})}^{-4}$ and *t*_1_ − *t*_0_ is small for short-living fast kernels. The dynamics of the separation motion was studied using AIA 304 Å data (Supplementary Section 1.3) whose field of view contains both ribbons that developed during the flare. *σ*(*s*_0,1_) = 0″.375 and *σ*(*t*_0,1_) = 3 s were taken as a quarter of the spatial and temporal resolution of AIA 304 Å observations.

### Impact of cadence and spatial resolution

The impact of instrumental cadence and spatial resolution on the detection of fast flare kernels was assessed by comparing the signatures of kernels in the time–distance diagram produced using the SJI 1,330 Å, AIA 304 Å and AIA 1,600 Å data. We also degraded the original SJI 1,330 Å time–distance diagram to the spatial resolution of the Transition Region and Coronal Explorer (TRACE^48^). This instrument, operating during 1998–2010, was in its flare mode capable of observing the Sun at a cadence of 2 s, comparable to the IRIS observations analysed here. Its spatial resolution was lower, with a pixel size of 0″.5 (~360 km), and the full width at half maximum of the core of its PSF was roughly 1″.25 (ref. ^49^). IRIS/SJI data were further degraded to the resolution of two ‘hypothetical instruments’, HI1 and HI2. Their pixel size and spatial resolution were set to be the same as those of IRIS, but their cadence was decreased to ~4 and ~8 s, respectively. Time–distance diagrams containing the AIA 304 Å, AIA 1,600 Å as well as degraded data were produced along the same artificial cut (Fig. 1g) that we used to study motions in the original SJI 1,330 Å observations.

The degradation of the original SJI observations to the resolution of TRACE consisted of decreasing their spatial resolution, followed by rebinning to a given pixel size. The spatial resolution was decreased by convolving SJI intensity maps with the Gaussian function via the ‘gauss_smooth’ function in IDL. The standard deviation of the Gaussian kernel was derived from the full width at half maximum of the TRACE PSF. The size of the smoothing boxcar was chosen to fit three standard deviations in each direction. Initial deconvolution of the SJI 1,330 Å time–distance diagram with the PSF of IRIS, followed by convolution using the proper PSF of TRACE, would be more precise, however the Gaussian approximation was sufficient for the purposes of our analysis. The decrease of the time resolution for HI1 and HI2 was achieved by selecting every second (cadence of ~4 s) and every fourth (cadence of ~8 s) frame in the SJI time series. It ought to be emphasized that this procedure omits smearing of signal in imagery, especially when acquired at longer exposure times. This degradation procedure accounts for the decrease of the instrumental resolution only but does not consider differences between the instrumental dynamic range, spectral content and other factors relevant for the potential visibility of kernels (Supplementary Section 1.6.2).

### Computer vision

The selection of kernel traces for fitting in time–distance diagrams (Fig. 2c,e) as well as determining the placement of the endpoints of the linear fits was performed manually. This approach may potentially introduce bias in assessing the properties of kernel motions when comparing time–distance diagrams from different instruments as well as the degraded data. Therefore, statistical analysis of traces of kernels’ motions was performed via an automatic computer vision algorithm for feature detection, effectively mitigating human-introduced errors.

#### Pre-processing of time–distance diagrams

Before the identification of kernel traces, the time–distance diagrams were convolved with the Sobel operator for the detection of edges along the vertical axis. This task was performed using the ‘sobel’ function available within the ‘ndimage’ library of SciPy^50^. This method was found to enhance the kernel traces while removing the background emission as well as the emission of stationary kernels and other brightenings. The processed time–distance diagrams were then adjusted to have equal saturation levels, between 0.01% and 20% of the maximal brightness, determined on a trial-and-error basis. The resulting time–distance diagrams were finally visualized using the bilinear filtering provided within the ‘matplotlib’ library. This was done to avoid pixelation of images leading to issues with the detection of traces via the computer vision algorithm.

#### Kernel identification via computer vision

Automatic identification of kernel traces in the pre-processed time–distance diagrams was performed using the feature detection algorithm provided within the OpenCV^51^ library. This process consists of several steps, preceded by image saturation between a user-defined minimal intensity and the maximal intensity ${I}_{\max }=255$ in the eight-bit grey-scale colour space. To investigate the performance of the method across a high dynamic range, we chose several values of the minimal intensity, corresponding to 10%, 30%, 50% and 90% of ${I}_{\max }$.

Intensity enhancements visible in the saturated time–distance diagrams are initially encompassed by contours, each enclosed within a rectangle corresponding to a separate feature. From all of the features identified by the method, we only considered those encompassed by rectangles larger than 500 pixels^2^ and with an aspect ratio >1.5 between the sides of the rectangle. These criteria ensured that features (1) imprinted by minor brightenings and (2) with small degree of elongation were excluded from the statistics. The features fulfilling these criteria are highlighted using magenta frames in the right column of Fig. 4. The numbers *n*_f_ of features (kernels traces) detected by the algorithm in selected time–distance diagrams are listed in Table 1 as a function of the minimal intensity threshold.

The feature detection algorithm also provides the orientation of the rectangles that we utilized to calculate kernel velocities. Because of the large number of features detected in certain time–distance diagrams, as well as because of the different orientations of kernel motions, we focused on the average absolute slipping velocities $\left\langle | {v}_{{\rm{slip}}}| \right\rangle$ together with their standard deviations ${\sigma }_{| {v}_{{\rm{slip}}}| }$. These values are listed in Table 1 and discussed in Supplementary Section 1.6.

### Temperature diagnostics

To study the temperature structure of the observed flare, we employed a regularized inversion method^52^ to calculate the DEM of flare plasma. Maps of DEMs in each spatial pixel in the field of view were recovered using imaging observations from AIA during the impulsive phase of the flare. Data from the 94 Å, 131 Å, 171 Å, 193 Å, 211 Å and 335 Å filter passbands were used, and the solutions were constrained to the temperature range between log(*T* [K]) = 5.6 and 7.4 with a temperature step of log(*T* [K]) = 0.1. DEM maps obtained in the temperature bins of log(*T* [K]) = 6.0−6.1, log(*T* [K]) = 6.5−6.6 and log(*T* [K]) = 7.0−7.1 are presented in Extended Data Fig. 3.

### The 3D MHD model of field line slippage

In this study, we revisit the existing 3D MHD experiment^12,28^ used to predict the existence of slipping reconnection. This zero-*β* (i.e. zero thermal pressure relative to magnetic pressure) resistive simulation was performed on a fixed non-uniform structured Cartesian mesh, using 191 × 161 × 170 points and with mesh intervals stretching by a factor of 10 away from the centre of the photospheric plane (*z* = 0). Viscous diffusion was modelled through a mesh-dependent filter, and reconnection was allowed through an explicit resistive term. Resistivity was fixed in space and time to suppress any acceleration effect typical for anomalous resistivity and hyperdiffusion schemes. The magnetic configuration of this numerical simulation contains two bipolar magnetic flux concentrations (P1 and N1, P2 and N2), an outer (P1 and N2) and a smaller inner (P2 and N1) bipole whose axis is oriented at an angle of 150° with respect to the outer bipole (Extended Data Fig. 6).

At the initiation of this time-dependent simulation (*t* = 0*t*_A_), the fields are potential and possess thin QSLs intersecting at a hyperbolic flux tube (HFT^36^). HFTs are preferable locations for field line reconnection in three dimensions, typically present in quadrupolar topologies. The squashing degree *Q* in the potential state peaks at *Q* = 6 × 10^8^ (ref. ^28^). Since the norm *N* of field line connectivity is roughly proportional to $\sqrt{Q}$ (ref. ^36^), its maximal value in this run is on the order of *N* = 10^4^. The evolution of the magnetic fields is driven by line-tied (at *z* = 0) translational and twisting motions of the positive polarity P2 of the inner bipole. This evolution is accompanied by the spontaneous development of current sheets within deforming QSLs, the strongest being found at the HFT. The driving is subsequently switched off, all velocities within the simulation are reset to zero and the simulation is allowed to relax. Despite the driving being turned off, some magnetic field lines are undergoing reconnection. These magnetic field lines are located within the QSLs, areas with high gradients of connectivity corresponding to diffusing current layers. This magnetic reconnection is manifested as the apparent slipping (sub-Alfvénic) and slip-running (super-Alfvénic) motions of field line footpoints along arc-shaped trajectories that correspond to the intersections of QSLs and the simulation domain. The process of field line slippage is further described in Supplementary Section 1.4 and is illustrated in Extended Data Fig. 6, produced using the TOPOTR code^20,53^.

## Supplementary information

Supplementary Information Supplementary Sections 1.1–1.6, Figs. 1 and 2 and References.

Supplementary Video 1 Animated version of Extended Data Fig. 1.

Supplementary Video 2 Animated version of Extended Data Fig. 2.

Supplementary Video 3 Animated version of Extended Data Fig. 5.

Supplementary Video 4 SDO/AIA 94 Å observations of the NOAA 13107 and 13105 active regions.

## Data Availability

The observations used in this study are publicly available and accessible via mission data archives. IRIS observations are available at https://iris.lmsal.com/data.html. SDO data can be accessed via JSOC at http://jsoc.stanford.edu/ajax/lookdata.html. The 3D MHD simulation outputs are available upon request.

## Code availability

Data were processed and visualized via libraries described in Methods. Time–distance diagrams were produced and visualized using a package available via https://github.com/JLo-Py/IDL_stackplot written in IDL. The Python script for running feature detection functions of the OpenCV library is available via https://github.com/JLo-Py/get_feature.

## References

[1] Zweibel, E. G. & Yamada, M. Magnetic reconnection in astrophysical and laboratory plasmas. *Annu. Rev. Astron. Astrophys.* **47**, 291–332 (2009).

[2] Nagai, F. & Emslie, A. G. Gas dynamics in the impulsive phase of solar flares. I Thick-target heating by nonthermal electrons. *Astrophys. J.* **279**, 896–908 (1984).

[3] Warren, H. P. & Warshall, A. D. Ultraviolet flare ribbon brightenings and the onset of hard X-ray emission. *Astrophys. J. Lett.* **560**, L87–L90 (2001).

[4] Acton, L. W. et al. Chromospheric evaporation in a well-observed compact flare. *Astrophys. J.* **263**, 409–422 (1982).

[5] Asai, A. et al. Flare ribbon expansion and energy release rate. *Astrophys. J.* **611**, 557–567 (2004).

[6] Qiu, J., Wang, H., Cheng, C. Z. & Gary, D. E. Magnetic reconnection and mass acceleration in flare-coronal mass ejection events. *Astrophys. J.* **604**, 900–905 (2004).

[7] Fletcher, L., Pollock, J. A. & Potts, H. E. Tracking of TRACE ultraviolet flare footpoints. *Solar Phys.* **222**, 279–298 (2004).

[8] Dudík, J. et al. Slipping magnetic reconnection during an X-class solar flare observed by SDO/AIA. *Astrophys. J.* **784**, 144 (2014).

[9] Li, T. & Zhang, J. Quasi-periodic slipping magnetic reconnection during an X-class solar flare observed by the Solar Dynamics Observatory and Interface Region Imaging Spectrograph. *Astrophys. J. Lett.* **804**, L8 (2015).

[10] Lörinčík, J., Aulanier, G., Dudík, J., Zemanová, A. & Dzifčáková, E. Velocities of flare kernels and the mapping norm of field line connectivity. *Astrophys. J.* **881**, 68 (2019).

[11] Priest, E. R. & Forbes, T. G. Magnetic flipping: reconnection in three dimensions without null points. *J. Geophys. Res.* **97**, 1521–1531 (1992).

[12] Aulanier, G., Pariat, E., Démoulin, P. & Devore, C. R. Slip-running reconnection in quasi-separatrix layers. *Solar Phys.* **238**, 347–376 (2006).

[13] Aulanier, G. et al. Slipping magnetic reconnection in coronal loops. *Science* **318**, 1588–1591 (2007). 18063789 10.1126/science.1146143

[14] Sun, X. et al. Hot spine loops and the nature of a late-phase solar flare. *Astrophys. J.* **778**, 139 (2013).

[15] Li, T. & Zhang, J. Slipping magnetic reconnection triggering a solar eruption of a triangle-shaped flag flux rope. *Astrophys. J. Lett.* **791**, L13 (2014).

[16] Li, T., Yang, K., Hou, Y. & Zhang, J. Slipping magnetic reconnection of flux-rope structures as a precursor to an eruptive X-class solar flare. *Astrophys. J.* **830**, 152 (2016).

[17] Pan, H., Gou, T. & Liu, R. Sigmoid formation through slippage of a single J-shaped coronal loop. *Astrophys. J.* **937**, 77 (2022).

[18] Dudík, J. et al. Slipping magnetic reconnection, chromospheric evaporation, implosion, and precursors in the 2014 September 10 X1.6-class solar flare. *Astrophys. J.* **823**, 41 (2016).

[19] Priest, E. R. & Démoulin, P. Three-dimensional magnetic reconnection without null points. 1. Basic theory of magnetic flipping. *J. Geophys. Res.* **100**, 23443–23464 (1995).

[20] Demoulin, P., Henoux, J. C., Priest, E. R. & Mandrini, C. H. Quasi-separatrix layers in solar flares. I. Method. *Astron. Astrophys.* **308**, 643–655 (1996).

[21] Pontin, D. I., Galsgaard, K., Hornig, G. & Priest, E. R. A fully magnetohydrodynamic simulation of three-dimensional non-null reconnection. *Phys. Plasmas* **12**, 052307 (2005).

[22] Demoulin, P., Bagala, L. G., Mandrini, C. H., Henoux, J. C. & Rovira, M. G. Quasi-separatrix layers in solar flares. II. Observed magnetic configurations. *Astron. Astrophys.* **325**, 305–317 (1997).

[23] Mandrini, C. H. et al. Evidence of magnetic reconnection from Hα, soft X-ray and photospheric magnetic field observations. *Solar Phys.* **174**, 229–240 (1997).

[24] Schmieder, B. et al. What is the role of magnetic null points in large flares? *Adv. Space Res.* **39**, 1840–1846 (2007).

[25] Lawrence, E. E. & Gekelman, W. Identification of a quasiseparatrix layer in a reconnecting laboratory magnetoplasma. *Phys. Rev. Lett.* **103**, 105002 (2009). 19792321 10.1103/PhysRevLett.103.105002

[26] Savcheva, A. et al. The relation between solar eruption topologies and observed flare features. I. Flare ribbons. *Astrophys. J.* **810**, 96 (2015).

[27] Zhao, J. et al. Hooked flare ribbons and flux-rope-related QSL footprints. *Astrophys. J.* **823**, 62 (2016).

[28] Aulanier, G., Pariat, E. & Démoulin, P. Current sheet formation in quasi-separatrix layers and hyperbolic flux tubes. *Astron. Astrophys.* **444**, 961–976 (2005).

[29] Aulanier, G., Janvier, M. & Schmieder, B. The standard flare model in three dimensions. I. Strong-to-weak shear transition in post-flare loops. *Astron. Astrophys.* **543**, A110 (2012).

[30] Janvier, M., Aulanier, G., Pariat, E. & Démoulin, P. The standard flare model in three dimensions. III. Slip-running reconnection properties. *Astron. Astrophys.* **555**, A77 (2013).

[31] Warmuth, A. & Mann, G. A model of the Alfvén speed in the solar corona. *Astron. Astrophys.* **435**, 1123–1135 (2005).

[32] Carmichael, H. A process for flares. *NASA Spec. Publ.* **50**, 451 (1964).

[33] Sturrock, P. A. Model of the high-energy phase of solar flares. *Nature* **211**, 695–697 (1966).

[34] Hirayama, T. Theoretical model of flares and prominences. I: evaporating flare model. *Solar Phys.* **34**, 323–338 (1974).

[35] Kopp, R. A. & Pneuman, G. W. Magnetic reconnection in the corona and the loop prominence phenomenon. *Solar Phys.* **50**, 85–98 (1976).

[36] Titov, V. S., Hornig, G. & Démoulin, P. Theory of magnetic connectivity in the solar corona. *J. Geophys. Res.(Space Phys.)* **107**, 1164 (2002).

[37] De Pontieu, B. et al. The Interface Region Imaging Spectrograph (IRIS). *Solar Phys.* **289**, 2733–2779 (2014). 10.1007/s11207-018-1364-8 PMC6394754 30880844

[38] Pesnell, W. D., Thompson, B. J. & Chamberlin, P. C. The Solar Dynamics Observatory (SDO). *Solar Phys.* **275**, 3–15 (2012).

[39] Lemen, J. R. et al. The Atmospheric Imaging Assembly (AIA) on the Solar Dynamics Observatory (SDO). *Solar Phys.* **275**, 17–40 (2012).

[40] Scherrer, P. H. et al. The Helioseismic and Magnetic Imager (HMI) investigation for the Solar Dynamics Observatory (SDO). *Solar Phys.* **275**, 207–227 (2012). 10.1007/s11207-018-1259-8 PMC6445534 31007294

[41] Testa, P. et al. Observing coronal nanoflares in active region moss. *Astrophys. J. Lett.* **770**, L1 (2013).

[42] Courrier, H., Kankelborg, C., De Pontieu, B. & Wülser, J.-P. An on orbit determination of point spread functions for the Interface Region Imaging Spectrograph. *Solar Phys.* **293**, 125 (2018). 10.1007/s11207-018-1347-9 PMC6190878 30393401

[43] SunPy Community. The SunPy project: open source development and status of the version 1.0 core package. *Astrophys. J.* **890**, 68 (2020).

[44] O’Dwyer, B., Del Zanna, G., Mason, H. E., Weber, M. A. & Tripathi, D. SDO/AIA response to coronal hole, quiet Sun, active region, and flare plasma. *Astron. Astrophys.* **521**, A21 (2010).

[45] Hunter, J. D. Matplotlib: a 2D graphics environment. *Comput. Sci. Eng.* **9**, 90–95 (2007).

[46] Lörinčík, J., Dudík, J., Aulanier, G., Schmieder, B. & Golub, L. Imaging evidence for solar wind outflows originating from a coronal mass ejection footpoint. *Astrophys. J.* **906**, 62 (2021).

[47] Dudík, J., Zuccarello, F. P., Aulanier, G., Schmieder, B. & Démoulin, P. Expanding and contracting coronal loops as evidence of vortex flows induced by solar eruptions. *Astrophys. J.* **844**, 54 (2017).

[48] Handy, B. N. et al. The transition region and coronal explorer. *Solar Phys.* **187**, 229–260 (1999).

[49] Golub, L. et al. A new view of the solar corona from the transition region and coronal explorer (TRACE). *Phys. Plasmas* **6**, 2205–2216 (1999).

[50] Virtanen, P. et al. SciPy 1.0: fundamental algorithms for scientific computing in Python. *Nat. Methods* **17**, 261–272 (2020). 32015543 10.1038/s41592-019-0686-2 PMC7056644

[51] Bradski, G. The OpenCV library. *Dr. Dobba’s J.* **120**, 122–125 (2000).

[52] Hannah, I. G. & Kontar, E. P. Multi-thermal dynamics and energetics of a coronal mass ejection in the low solar atmosphere. *Astron. Astrophys.* **553**, A10 (2013).

[53] Pariat, E. & Démoulin, P. Estimation of the squashing degree within a three-dimensional domain. *Astron. Astrophys.* **541**, A78 (2012).

