# Supplementary material for: Observation of super-Alfvénic slippage of reconnecting magnetic field lines on the Sun
Source: Nat Astron. 2024 Oct 18;9(1):45–54. doi: 10.1038/s41550-024-02396-4 (PMC11757146; doi:10.1038/s41550-024-02396-4)
Supplement: Supplementary file 1 — Supplementary Sections 1.1–1.6, Figs. 1 and 2 and References. [file 41550_2024_2396_MOESM1_ESM.pdf]

nature astronomy

Supplementary information https://doi.org/10.1038/s41550-024-02396-4

Observation of super-Alfvénic slippage of reconnecting magnetic field lines on the Sun

In the format provided by the authors and unedited

1 Supplementary information

1.1 Magnetic topology of the event

The NOAA active region (AR) 13107, where the flare occurred, was located in a close vicinity to the NOAA AR13105. **Extended Data Fig. 2** consists of a B_{LOS} map observed by HMI overlaid by snapshots from the AIA 131 Å (cyan) and AIA 171 Å (yellow) and, as such, presents context observations of these active regions (see also Supplementary Video 2). The AR13107 is located to the south-east (left), while the AR13105 to the north-west (right). Both ARs are bipolar, with the negative (N1, N2) leading and positive (P1, P2) trailing polarities. The overall quadrupolar configuration is closely reminiscent of the original 3D MHD model of the slipping reconnection[12,28] (**Methods**).

AIA 171 Å observations reveal four dominant sets of coronal loops with distinct magnetic connectivities in these ARs. The first two sets connect the positive and negative polarity flux concentrations (P1 & N1, P2 & N2) within the two respective ARs. Another set of field lines can be found between the N1 and P2 polarities of the AR13107 and AR13105, respectively, composing an inner bipole in the middle of the FOV. The last set of field lines is represented by the large, overarching coronal loops connecting the outer polarities of the two ARs, P1 in the AR13107 and N2 in the AR13105. These four sets of loops are separated by a seemingly vacant, low-emission AIA 171 Å region with an X-like morphology visible in the upper part of the field-of-view (FOV). This feature is reminiscent of the HFT, the reconnection site in quadrupolar topologies also present in the 3D MHD model investigated **in Methods**. AIA 94 Å observations of roughly 7 MK emission (Supplementary Video 4) provide evidence that the two active regions exhibited correlated activity in the time period leading to the flare, as well as during the subsequent flare which set on in the same region after 07:00 UT. The observed evolution ascertains this feature as the HFT, the primary site of reconnection in this topology.

1.2 Alfvén speed in the observed environment

Flare kernels slipping along the ribbon are interpreted (**Introduction**) to be footpoints of magnetic field lines undergoing slipping reconnection and the associated energy release. While the bulk of kernel emission originates in the solar transition region and the chromosphere, the field line slippage occurs throughout the associated QSLs in the corona. Whether the slippage is sub- or super-Alfvénic therefore depends on the magnitude of the Alfvén speed c_A in the corona. The c_A is equal to $B/\sqrt{\mu\rho}$, where B is the magnetic field strength, ρ is the plasma density, and μ is the magnetic permeability. The determination of c_A is at present limited by the lack of ability to accurately measure coronal magnetic fields. Nevertheless, several studies inferred average fields B in the corona of the order of 10^1 G[54–57]. For $|B| < 100$ G and density $\rho = 5.10^8 - 10^9$ cm^{-3}, typical for active region conditions, we obtain c_A ranging between a few hundred up to ≈ 1200 km s^{-1}, in agreement with previous measurements[31]. Note that larger $|B|$ values of several 10^2 G can occur in larger flares[58,59]; while smaller $|B| < 10$ G are typical for very long coronal loops[54]. Both situations are not relevant to our small flare occurring in active regions 13107 and 13105 with dispersed magnetic polarities (**Extended Data Fig. 2**, **Extended Data Fig. 4**).

1.3 Ribbon separation

As mentioned in **Results**, the eastern ('E') and western ('W') ribbons exhibited slow separation (**Introduction**), motion of the entire ribbon structures in the direction away from the inversion line. The separation motion is demonstrated in **Extended Data Fig. 4**a, where we plotted 500 DN intensity contours observed in the AIA 304 Å passband in snapshots from ≈06:15, 06:25, and 06:40 UT (yellow, cyan, red). The contours detail the evolving ribbons during the initiation of the flare as well as during its impulsive and gradual phases, respectively. They are overlaid on the SDO/HMI B_{LOS} magnetograms saturated to ±1000 G and observed at ≈06:25 UT. A comparison between the AIA and HMI observations reveals that the E ribbon, which elongated roughly in the north-south direction, was spatially coincident with the positive polarity flux concentrations P1 of the AR13107. The W ribbon, that elongated in the east-west direction, formed in the negative polarity flux concentrations N1 of the same active region.

The directions of the separation motions of the two ribbons were not parallel, contrary to the ribbon dynamics often reported in the literature. While the E ribbon exhibited the separation motion towards the east direction, the W ribbon moved southward. These directions are indicated by the green arrows in **Extended Data Fig. 4**a, which we also utilized to construct the time-distance diagrams (**Extended Data Fig. 4**b, c) employed to measure the separation speeds. The separation of the W ribbon commenced after ≈06:14 UT and lasted till ≈06:28. The linear fit to the ribbon motion, showed using the blue dotted line in **Extended Data Fig. 4**b, corresponds to $v_\perp = 2.1 \pm 0.5$ km s^{-1}. The E ribbon exhibited a faster separation roughly between 06:18 – 06:25 UT at $v_\perp$ of 4.6 ± 1.0 km s^{-1} (**Extended Data Fig. 4**c).

1.4 Field line slippage in 3D MHD model

Extended Data Fig. 6 presents the apparent slipping motion of magnetic field lines in the 3D MHD simulation (**Methods**) at four instants ($t = 0, 3, 10, 30\, t_{\mathrm{A}}$) after the driving of the boundary motions had been switched off. **Extended Data Fig. 6**a – d provides a detailed view of the field line slippage along concentrations of the vertical electric current $J_{z=0}$, associated to flare

ribbons[60]. These concentrations are encompassed by pink and cyan contours corresponding to $J_{z=0} = \pm 0.3$ (in code units). The viewing geometry in **Extended Data Fig.** 6e – h mimics that of the observed event.

The positive (P1, P2) and the negative (N1, N2) polarity magnetic flux concentrations of the two bipoles included in the simulation are shown in white and black, respectively, and are saturated to $B_{z=0} = \pm 25$ (in code units). Their distribution (P1, N1, P2, N2, from left to right in **Extended Data Fig.** 6e – h) is similar to the magnetic topology in which the flare analyzed here was observed (**Extended Data Fig.** 2, **Supplementary Information Section 1.1**). The flux concentrations are interconnected by four ensembles of field lines colored using the orange, green, and magenta colors. The low-lying orange field lines connect the positive and the negative polarities of the two seemingly independent bipoles (P1 & N1, P2 & N2) on the left- and right-hand side (**Extended Data Fig.** 6a). These field lines are overlaid by orange field lines connecting the outer polarities (P1 & N2) and separated by magenta field lines connecting the polarities of the inner bipole (P2 & N1). The magenta and green field lines, a subset of the field lines connecting the outer bipole, are located in the vicinity of a QSL (and current layers therein) and therefore slip-reconnect. The footpoints of these two sets of field lines, located in the positive polarity flux concentrations P1 and P2, have been fixed to demonstrate their slippage between the two negative polarity flux concentrations N1 and N2.

The negative polarity footpoints of the magenta and green field lines are initially ($t_A = 0$) rooted in the N1 and N2 flux concentrations, respectively, in the far ends of the QSL. Subsequently, as demonstrated in **Extended Data Fig.** 6f, g ($t_A = 3, 10$), the footpoints of the green field lines slip towards the negative polarity N1, as they exchange their connectivities with the magenta field lines slipping towards the negative polarity N2. In the final snapshot ($t_A = 30$), the field line slippage ended and the connectivities of the magenta and the green field lines have been completely exchanged. The negative polarity footpoints of the magenta and green field lines can be found in the N2 and N1, respectively, as opposed to their initial locations (c.f. **Extended Data Fig.** 6e, **Extended Data Fig.** 6h).

Even though the revisited vintage 3D MHD simulation[12,28] does not emulate the detailed magnetic environment where the flare occurred, the principal characteristics of the modeled slippage are consistent with our observations. The observed analogy to the slippage of the green field lines described above is the apparent slipping motion of the kernels detailed **in Results.** Both the observed and simulated motions occurred in the same negative polarity N1 of the left bipole (AR13107) of the overall quadrupolar topology. While the majority of the kernel motions were observed in the west-east direction, the simulated slippage within N1 was primarily directed between the top and the bottom of the domain (from the viewpoint of **Extended Data Fig.** 6e – h). This difference can be attributed to the east-west elongation of the N1 flux concentrations of the AR13107, likely affecting the morphology of QSL footprints and thus the reconnection along them.

Our data are also indicative of kernel slippage in the opposite direction (from the east to the west), consistent with the motion of the magenta field lines within N1. The most notable counter-propagating kernel is visible between ≈06:19 – 06:20 UT in Supplementary Video 3, slipping at velocity of about 40 km s^{-1}. Although some counter-propagating kernels can be identified in the SJI 1330 Å observations, their velocities are low, for which they do not appear in the LRR-processed images (Fig. 2). Our results are in agreement with previous observational studies[8,9,61] reporting on slipping motions in a preferential direction. This may be attributed to the asymmetric distribution of the magnetic flux within the two active regions, or to the change in lengths of the reconnecting loops. **Extended Data Fig.** 6 indicates that as result of the slippage, the green field lines were shrinking, while the magenta ones were increasing their length. This prolongation could in turn lead to the decrease of loop density and hence emission measure, making the loops less visible.

1.5 Slipping velocities and their variations

The separation of the field line footpoints in **Extended Data Fig.** 6 is indicative of variations of v_{slip} along the footprints of current concentrations ($J_{z=0}$). In the simulation, the footpoints of the field lines are close one to another in the N1 and N2 polarities where the magnetic field is stronger and the $J_{z=0}$ contours are larger (**Extended Data Fig.** 6e, h). The slippage therein occurs in the slow, sub-Alfvénic regime. The footpoint separation (and hence v_{slip}) increases along narrow $J_{z=0}$ concentrations between the N1 and N2 polarities where the field is weak (**Extended Data Fig.** 6f, g).

Motivated by this prediction, we produced a time-distance diagram detailing the distribution of negative-polarity flux concentrations observed by HMI along the ribbon via the same cut used to study v_{slip} (Supplementary Fig. 1**a**). The white and black colors correspond to $B_{LOS} = 0$, and -1000 G, respectively, and the cyan, purple, and blue contours delineate $B_{LOS} = -10$, -100, and -300 G. The same contours are plotted in Supplementary Fig. 1b over a section of the original SJI 1330 Å time-distance diagram (Fig. 2a). According to this figure, the observed kernel traces tend to be more vertical (relatively-higher v_{slip}) in weak-field regions where $|B_{LOS}| < 100$ G. The emission coincident with strong-field regions ($|B_{LOS}| > 300$ G, e.g. at > 27" of the cut) appears more horizontal (stationary). The green arrows in panel b indicate two traces imprinted by fast kernels emanating from weak-field regions. These traces become near-horizontal (arrow Nr. 1) or disappear (arrow Nr. 2) as the kernels enter the strong-field regions, in agreement with previous studies[10,61]. On the other hand, the kernel traces highlighted using the red arrows appear unrelated to the underlying field. The arrow Nr. 3 points toward the slow ($v_{slip} \approx 40$ km s^{-1}) counter-propagating kernel (**Supplementary Information Section 1.4**) in a weak-field region. The arrow Nr. 4 highlights

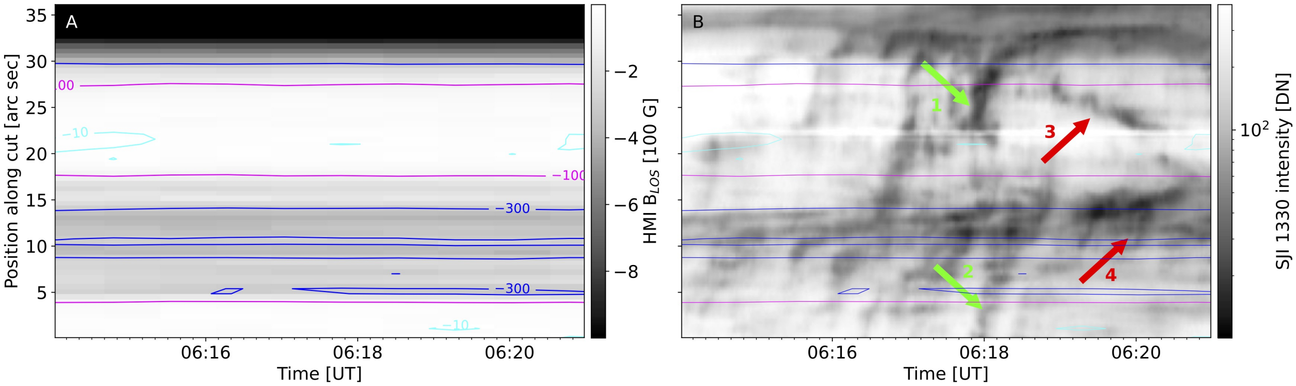

Supplementary Fig. 1. Comparison between the spatio-temporal distribution of $B_{\rm LOS}$ and signatures of kernel motions. Panel a presents time-distance diagram produced via HMI $B_{\rm LOS}$ observations along the ribbon saturated between $B_{\rm LOS} = 0\,$G (white) and $B_{\rm LOS} = -1000\,$G (black). The cyan, purple, and blue contours correspond to $B_{\rm LOS} = -10, -100, -100\,$G. The same contorus are plotted in panel b, which depicts a portion of the original SJI 1330 Å time-distance diagram plotted in Fig. 2a. The numbered green and red arrows refer to signatures of kernel motions discussed in **Supplementary Information Section 1.5**.

a trace of a fast kernel within a strong-field region. This brief analysis suggests that the anti-correlation between the two quantities likely isn't absolute, and $v_{\rm slip}$ as well as its variations are also guided by different parameters.

Beyond the relation to the magnitude of the surface magnetic field, 3D MHD extensions to the standard flare model[30] shown that $v_{\rm slip}$ strongly correlates with the norm N, measure of distortion of field line connectivity. The analysis of variations of $v_{\rm slip}$ of a selected field line presented in Fig. 7 therein shows that the super-Alfvénic slippage occurs through a region where $N = 10^2 - 10^3$. Our upper estimate of $N = 24 - 577$ obtained via the measured $v_{\rm slip}$ (**Results**) and $v_{\perp}$ (**Supplementary Information Section 1.3**) falls within this range, indicating that the fastest kernel motions were consistent with high gradients of connectivity and vice versa.

1.6 Signatures in observations of different instruments

1.6.1 Statistical analysis

Perhaps the most important outcome of the statistical analysis of kernel signatures in time-distance diagrams via the computer vision method concerns the slipping velocities. As briefly mentioned **in Results**, we found a good correspondence between $v_{\rm slip}$ obtained from the orientation of the features in the SJI 1330 Å time-distance diagram processed via the Sobel operator and the manual fitting of kernel traces in the LRR-processed image. The range of $v_{\rm slip}$ deduced via the computer vision technique is broad, similar to that obtained via the fitting procedure (Fig. 2c, e). The majority of the detected kernels exhibited slippage in one direction, along the ribbon from west to east (see also **Supplementary Information Section 1.4**). About $20-40\%$ of the kernel traces are found to be signatures of counter-propagating motions, though this fraction is dependent on the image saturation and thus carries a relatively high uncertainty. From now on we thus focus on the absolute values of $v_{\rm slip}$.

The histogram of $|v_{\rm slip}|$ plotted in Supplementary Fig. 2c demonstrates that the distribution of $|v_{\rm slip}|$ is far from Gaussian, resulting in high $\sigma_{|v_{\rm slip}|}$ (see Table 1). Table 1 next shows that the number of features $n_{\rm f}$ is the highest in time-distance diagrams saturated to I = 10% of $I_{\rm max}$, containing kernel traces with a broad range of intensities. For example, $n_{\rm f}$ detected in SJI 1330 Å data considering the 10% image saturation is by a factor of ≈ 4 larger than $n_{\rm f}$ identified in the same diagram saturated to 90% of $I_{\rm max}$. Only a fraction of kernels thus reach high intensities, while most of them are faint. Our analysis indicates that the brightest kernels are more likely to be detected in both high- and low- resolution data. We found a factor of ≈ 2.6 difference between $n_{\rm f}$ resolved at relatively higher (SJI 1330 Å) and relatively lower (AIA 304 Å) cadence in diagrams saturated to 90% of $I_{\rm max}$, compared to a factor ≈ 4.5 difference in the same images saturated to 10% of $I_{\rm max}$. The brightest kernels resolved by IRIS are however still considerably faster, with $\langle|v_{\rm slip}|\rangle = 1,122\,{\rm km\,s^{-1}}$, than those resolved in the AIA 304 Å image ($\langle|v_{\rm slip}|\rangle = 96\,{\rm km\,s^{-1}}$). This result holds in general, as $\langle|v_{\rm slip}|\rangle$ detected in the SJI time-distance diagram are by at least one and two orders of magnitude higher than those resolved in the AIA 304 Å and 1600 Å time-distance diagrams, respectively. The detection of kernels with $|v_{\rm slip}|$ exceeding $10^3\,{\rm km\,s^{-1}}$ would also be possible in data of the hypothetical instrument HI1 with a $\approx 4\,$s cadence, when $\sigma_{|v_{\rm slip}|}$ is taken into the account. Further twofold decrease of the time resolution, implemented for HI2, would impede such measurements.

1.6.2 *Why were fast kernels elusive in previous observations?*

The kernel slipping velocities surpassing 10^3 km s^{-1} are the key observable of this study, made possible by the high resolution of IRIS (**Supplementary Information Section 1.6.1**). One might wonder why slipping velocities of this order of magnitude have not been reported in the past, with previous observatories such as TRACE and GREGOR capable of observing at a cadence comparable to IRIS. For instance, slipping kernels studied in the GREGOR telescope Ca II line observations of a small C-class flare from 2014 November 7, acquired at a 1 s cadence[62], were found to be very slow with $v_{\mathrm{slip}} < 12$ km s^{-1}. The GREGOR observations were however limited by a small FOV of only a few arc seconds. A super-Alfvénic kernels would traverse this FOV very rapidly, and would be difficult to detect. The kernel brightenings observed at a 2 s cadence in the 1600 Å passband of TRACE during a large M8.5-class flare from 2002 July 17[7] were also relatively slow, not exhibiting dynamics surpassing 120 km s^{-1}. We revisited this dataset to rule out possible differences of v_{slip} due to different measurement methods but did not find evidence for faster kernels at all, in agreement with authors' findings. It ought to be noted that numerous TRACE snapshots were affected by image saturation, a consequence of lower dynamic range of the instrument's CCD camera. Apart from the issues with the image saturation and the size of the FOV, there are several additional factors that might explain the absence of evidence for fast kernel motions.

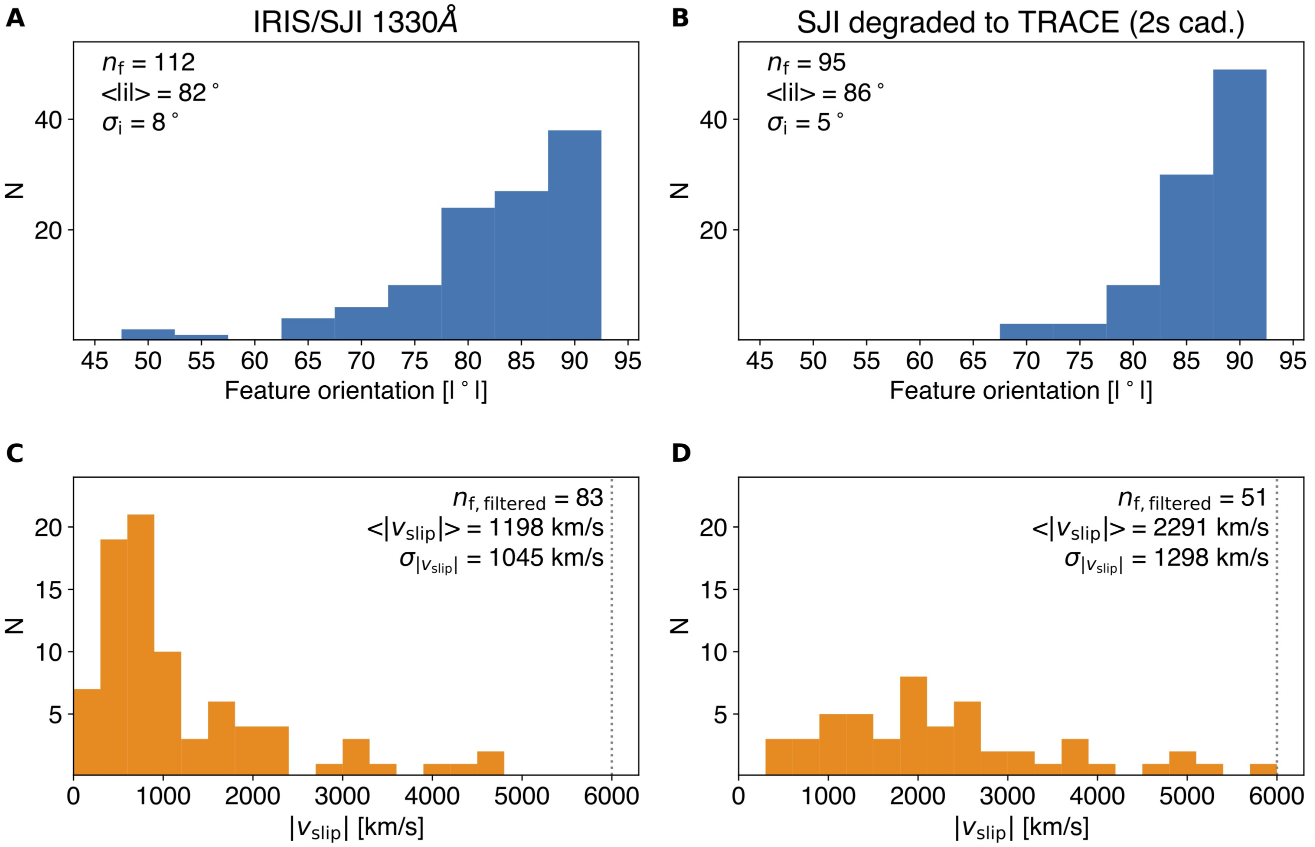

Supplementary Fig. 2. Distribution of feature orientations and slipping velocities. Histograms in panels a, b detail the distribution of orientations of features detected by the computer vision algorithm for the SJI 1330 Å data (panel a) and the SJI data degraded to the resolution of TRACE (panel b). Panels b, c show the distribution of the slipping velocities $|v_{\mathrm{slip}}|$ calculated using these orientations. The mean values $\langle |v_{\mathrm{slip}}| \rangle$ as well the standard deviations ($\sigma_{|v_{\mathrm{slip}}|}$) of these quantities are displayed in each plot. n_{f} is the number of samples considered in each histogram. Values of $|v_{\mathrm{slip}}|$, surpassing the maximal detectable velocity of 6,000 km s^{-1} (**Methods,** grey dotted line in panels c, d), have been excluded from the statistics.

First, these observations were carried-out in a different spectral range than the IRIS observations analyzed here. The C II lines, dominating the narrow-band SJI 1330 Å passband, (**Methods**) are partially formed in the transition region[63,64] characterized by a very rapid response to flare heating[65]. The wavelength response of the 1600 Å passband of TRACE was much broader, spanning hundreds of Ångstroms. A major contributor to this channel was the UV continuum[48] forming at much lower temperatures in the low-lying regions of solar photosphere and chromosphere[66] whose response to heating might not be as prompt as that of the transition region. The 1600 Å passband of TRACE was also sensitive to the emission of C I and Fe II[48]

lines forming in the chromosphere[67], just like the Ca II line observed by GREGOR. By inspecting the SJI 2796 Å dataset we found that only the brightest kernels are visible in chromospheric (Mg II k) imagery, missing a wealth of information contained in the SJI 1330 Å data.

In addition to the time resolution, dynamic range, and spectral content of the observations, the detection of kernels as well as precise determination of their dynamics relies upon the spatial resolution. High spatial resolution is essential not only for the detection of small-scale kernel brightenings, but also for the correct determination of their dynamics. Table 1 summarizes results of our simple experiment consisting of the degradation of the original SJI 1330 Å data to the spatial resolution of TRACE observing in a flare mode (2 s cadence). The degradation of the spatial resolution alone does not lead to a significant decrease of n_f between the two images (n_f = 112 vs. 95 for 10% of I_{max}). However, considering the same image saturation, $\langle|v_{slip}|\rangle$ calculated using the orientation of features detected in the TRACE time-distance diagram increased to 2,291 km s^{-1}, compared to 1,198 km s^{-1} in the original SJI 1330 Å image. This increase is a data artifact introduced by the image degradation. Due to the relatively-lower resolution of the degraded data, the motion of these structures can no longer be distinguished from their instantaneous appearance, leading to an apparent increase of their mean velocity.

Supplementary Fig. 2 presents histograms detailing the distributions of the absolute value of feature orientation (Supplementary Fig. 2a, b) and corresponding $|v_{slip}|$ (Supplementary Fig. 2c, d) in the original SJI 1330 Å (left column) and the degraded (right column) time-distance diagrams saturated to 10% of I_{max}. The number of features n_f in the sample, as well as the $\langle|v_{slip}|\rangle$ and the standard deviations $\sigma(|v_{slip}|)$ are indicated in each histogram. From the statistics of $|v_{slip}|$ we excluded those exceeding the maximal velocity detection limit of 6,000 km s^{-1} (grey dotted line, **Methods**), most of which correspond to feature orientations $i \rightarrow 90°$ (instantaneous appearance). Supplementary Fig. 2a, b show that the data degradation led to an overall increase of the orientation of the detected features. The increase of feature orientations is translated into a lower sample of $|v_{slip}| < 10^3$ km s^{-1} (c.f. Supplementary Fig. 2c, d) while the entire distribution shifts toward higher velocities, from $|v_{slip}| = 144 - 4,575$ km s^{-1} to $|v_{slip}| = 572 - 5,914$ km s^{-1} in the degraded image.

In conclusion, the discovery of the super-Alvénic field line slippage presented in this study is a result of a complex interplay of observational and physical factors. Of equal importance is the availability of newly-designed high-cadence IRIS observations, as a result of prioritizing these observations in flare-prone active regions whenever possible by the IRIS team. Future analyses focused on flares of different magnitudes, triggered within diverse magnetic environments, and observed at a very high time resolution should provide further insights into physics of super-Alfvénic slipping motions, as well as the frequency of their occurrence.

References for Supplementary Information

7. Fletcher, L., Pollock, J. A. & Potts, H. E. Tracking of TRACE Ultraviolet Flare Footpoints. *Sol. Phys.* **222**, 279–298, DOI: 10.1023/B:SOLA.0000043580.89730.4d (2004).

8. Dudík, J. *et al.* Slipping Magnetic Reconnection during an X-class Solar Flare Observed by SDO/AIA. *The Astrophys. J.* **784**, 144, DOI: 10.1088/0004-637X/784/2/144 (2014). 1401.7529.

9. Li, T. & Zhang, J. Quasi-periodic Slipping Magnetic Reconnection During an X-class Solar Flare Observed by the Solar Dynamics Observatory and Interface Region Imaging Spectrograph. *The Astrophys. J. Lett.* **804**, L8, DOI: 10.1088/2041-8205/804/1/L8 (2015). 1504.01111.

10. Lörinčík, J., Aulanier, G., Dudík, J., Zemanová, A. & Dzifčáková, E. Velocities of Flare Kernels and the Mapping Norm of Field Line Connectivity. *The Astrophys. J.* **881**, 68, DOI: 10.3847/1538-4357/ab298f (2019). 1906.01880.

12. Aulanier, G., Pariat, E., Démoulin, P. & Devore, C. R. Slip-Running Reconnection in Quasi-Separatrix Layers. *Sol. Phys.* **238**, 347–376, DOI: 10.1007/s11207-006-0230-2 (2006).

28. Aulanier, G., Pariat, E. & Démoulin, P. Current sheet formation in quasi-separatrix layers and hyperbolic flux tubes. *Astron. Astrophys.* **444**, 961–976, DOI: 10.1051/0004-6361:20053600 (2005).

30. Janvier, M., Aulanier, G., Pariat, E. & Démoulin, P. The standard flare model in three dimensions. III. Slip-running reconnection properties. *Astron. Astrophys.* **555**, A77, DOI: 10.1051/0004-6361/201321164 (2013). 1305.4053.

31. Warmuth, A. & Mann, G. A model of the Alfvén speed in the solar corona. *Astron. Astrophys.* **435**, 1123–1135, DOI: 10.1051/0004-6361:20042169 (2005).

48. Handy, B. N. *et al.* The transition region and coronal explorer. *Sol. Phys.* **187**, 229–260, DOI: 10.1023/A:1005166902804 (1999).

54. Jain, R. & Mandrini, C. H. The relationship between magnetic field strength and loop lengths in solar coronal active regions. *Astron. Astrophys.* **450**, 375–381, DOI: 10.1051/0004-6361:20053619 (2006).

55. Peter, H., Warnecke, J., Chitta, L. P. & Cameron, R. H. Limitations of force-free magnetic field extrapolations: Revisiting basic assumptions. *Astron. Astrophys.* **584**, A68, DOI: 10.1051/0004-6361/201527057 (2015). 1510.04642.

56. Froment, C. *et al.* Long-period Intensity Pulsations in Coronal Loops Explained by Thermal Non-equilibrium Cycles. *The Astrophys. J.* **835**, 272, DOI: 10.3847/1538-4357/835/2/272 (2017). 1701.01309.

57. Brooks, D. H., Warren, H. P. & Landi, E. Measurements of Coronal Magnetic Field Strengths in Solar Active Region Loops. *The Astrophys. J. Lett.* **915**, L24, DOI: 10.3847/2041-8213/ac0c84 (2021). 2106.10884.

58. Gary, D. E. *et al.* Microwave and Hard X-Ray Observations of the 2017 September 10 Solar Limb Flare. *The Astrophys. J.* **863**, 83, DOI: 10.3847/1538-4357/aad0ef (2018). 1807.02498.

59. Kuridze, D. *et al.* Mapping the Magnetic Field of Flare Coronal Loops. *The Astrophys. J.* **874**, 126, DOI: 10.3847/1538-4357/ab08e9 (2019). 1902.07514.

60. Janvier, M. *et al.* Electric Currents in Flare Ribbons: Observations and Three-dimensional Standard Model. *The Astrophys. J.* **788**, 60, DOI: 10.1088/0004-637X/788/1/60 (2014). 1402.2010.

61. Lörinčík, J., Dudík, J. & Polito, V. Blueshifted Si IV 1402.77 Å Line Profiles in a Moving Flare Kernel Observed by IRIS. *The Astrophys. J.* **934**, 80, DOI: 10.3847/1538-4357/ac78e2 (2022).

62. Sobotka, M. *et al.* Slipping reconnection in a solar flare observed in high resolution with the GREGOR solar telescope. *Astron. Astrophys.* **596**, A1, DOI: 10.1051/0004-6361/201527966 (2016). 1605.00464.

63. Rathore, B., Pereira, T. M. D., Carlsson, M. & De Pontieu, B. The Formation of Iris Diagnostics. VIII. Iris Observations in the C II 133.5 nm Multiplet. *The Astrophys. J.* **814**, 70, DOI: 10.1088/0004-637X/814/1/70 (2015). 1510.04845.

64. Sainz Dalda, A. & De Pontieu, B. Chromospheric thermodynamic conditions from inversions of complex Mg II h & k profiles observed in flares. *Front. Astron. Space Sci.* **10**, 1133429, DOI: 10.3389/fspas.2023.1133429 (2023). 2211.05459.

65. Tandberg-Hanssen, E., Reichmann, E. & Woodgate, B. Behavior of Transition Region Lines during Impulsive Solar Flares. *Sol. Phys.* **86**, 159–171, DOI: 10.1007/BF00157184 (1983).

66. Kleint, L., Heinzel, P., Judge, P. & Krucker, S. Continuum Enhancements in the Ultraviolet, the Visible and the Infrared during the X1 Flare on 2014 March 29. *The Astrophys. J.* **816**, 88, DOI: 10.3847/0004-637X/816/2/88 (2016). 1511.04161.

67. Graham, D. R. *et al.* Spectral Signatures of Chromospheric Condensation in a Major Solar Flare. *The Astrophys. J.* **895**, 6, DOI: 10.3847/1538-4357/ab88ad (2020). 2004.05075.
